# Diethyl 2,5-bis­[(*E*)-2-furylmethyl­ene­amino]thio­phene-3,4-dicarboxyl­ate

**DOI:** 10.1107/S1600536808006612

**Published:** 2008-03-14

**Authors:** Stéphane Dufresne, W. G. Skene

**Affiliations:** a Department of Chemistry, University of Montreal, CP 6128, succ. Centre-ville, Montréal, Québec, Canada H3C 3J7

## Abstract

The title compound, C_20_H_18_N_2_O_6_S, crystallizes as two independent mol­ecules that are disposed about a pseudo-inversion center (1/2, 1/4, 1/8). The mean planes of the two terminal furyl rings are twisted with respect to the central thio­phene ring by 7.33 (4) and 21.74 (5)° in one mol­ecule, and by 6.91 (4) and 39.80 (6)° in the other.

## Related literature

For general background, see: Dufresne *et al.* (2007 ▶). For related literature, see: Dufresne *et al.* (2006 ▶). For compounds crystallizing with two independent mol­ecules in the space groups *Pca*2_1_ and *Pna*2_1_, disposed about a pseudo-inversion center, see: Marsh *et al.* (1998 ▶).
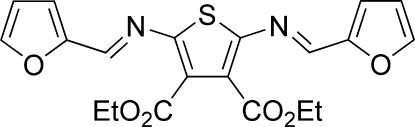


## Experimental

### 

#### Crystal data

C_20_H_18_N_2_O_6_S
*M* *_r_* = 414.42
Orthorhombic, 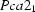

*a* = 8.2540 (3) Å
*b* = 10.1578 (3) Å
*c* = 46.087 (2) Å
*V* = 3864.0 (2) Å^3^
*Z* = 8
Cu *K*α radiation
μ = 1.85 mm^−1^
*T* = 220 (2) K
0.28 × 0.23 × 0.14 mm

#### Data collection

Bruker SMART 2000 diffractometer
Absorption correction: multi-scan (*SADABS*; Sheldrick, 1996 ▶) *T*_min_ = 0.640, *T*_max_ = 0.781
45562 measured reflections
7392 independent reflections
7164 reflections with *I* > 2σ(*I*)
*R*_int_ = 0.030

#### Refinement

*R*[*F* ^2^ > 2σ(*F* ^2^)] = 0.032
*wR*(*F* ^2^) = 0.087
*S* = 1.03
7392 reflections
527 parameters
1 restraint
H-atom parameters constrained
Δρ_max_ = 0.25 e Å^−3^
Δρ_min_ = −0.21 e Å^−3^
Absolute structure: Flack (1983 ▶), 3551 Friedel pairs
Flack parameter: 0.021 (10)

### 

Data collection: *SMART* (Bruker, 1999 ▶); cell refinement: *SAINT* (Bruker, 1999 ▶); data reduction: *SAINT*; program(s) used to solve structure: *SHELXS97* (Sheldrick, 2008 ▶); program(s) used to refine structure: *SHELXL97* (Sheldrick, 2008 ▶); molecular graphics: *ORTEP-3* (Farrugia, 1997 ▶); software used to prepare material for publication: *UdMX* (Marris, 2004 ▶).

## Supplementary Material

Crystal structure: contains datablocks I, global. DOI: 10.1107/S1600536808006612/ng2429sup1.cif

Structure factors: contains datablocks I. DOI: 10.1107/S1600536808006612/ng2429Isup2.hkl

Additional supplementary materials:  crystallographic information; 3D view; checkCIF report

## Figures and Tables

**Fig. 1.**
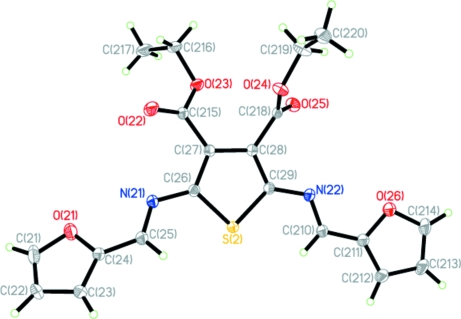
ORTEP representation of the two different molecules of (I) with the numbering scheme adopted (Farrugia 1997). Ellipsoids drawn at 30% probability level.

**Fig. 2.**
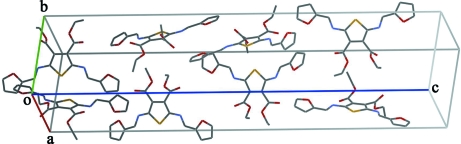
The three-dimensional network demonstrating the closed packing in the lattice.

**Fig. 3.**
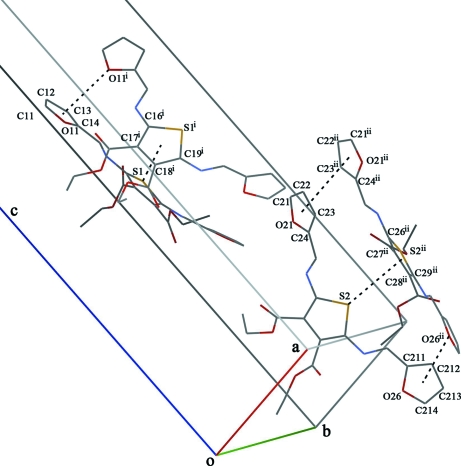
Supramolecular structure showing the intermolecular π-stacking giving the structural arrangement. Dashed lines indicate the π-stacking. [Symmetry codes: (i) 1/2 + x, -y, z; (ii) 1/2 + x, 1 - y, z.]

## supplementary crystallographic information

### Comment

The molecule (I) was prepared as a result of our ongoing research of conjugated azomethines for electronic devices. The crystal structure of (I) confirmed that the compound consisted of a central thiophene capped by two terminal furans that are connected by two azomethine bonds. Even though two isomers are possible, only the more stable E isomer was confirmed by the resolved structure. The chemical structure occurs eight times in the Pca2_1_ lattice as seen in Figure 2 with two different molecules of (I) per cell disposed near a false inversion center at 1/2, 1/4, 1/8. (Marsh *et al.*, 1998) Neither solvent nor counter-ions were found in the closed-packed stacking.

A major point of interest is the azomethine bond. The measured imine bond lengths for C14—C15, N11—C15 and N11—C16 are 1.429 (2), 1.287 (2) and 1.375 (2) Å, respectively. The bond distances are comparable to an all thiophene bisazomethine analogue (Dufresne *et al.*, 2006) whose analogous lengths are 1.441 (4), 1.272 (3) and 1.388 (3) Å.

The mean plane angles described by all three heterocycles of (I) are not entirely coplanar. The mean plane angles of the terminal furans are twisted 7.33 (4)° and 21.74 (5)° for one molecule of (I) with respect to the central thiohene. Similarly, the mean planes are twisted by 6.91 (4)° and 39.80 (6)° for the second molecule found in the lattice. Meanwhile, the average mean plane angles for the analogous all thiophene azomethine are 9.04 (4)° and 25.07 (6)°.

Interestingly, the three-dimensional network of (I) is very different than for its all thiophene analogue in which all the molecules are linear and aligned in one direction. Since no traditional hydrogen bonding occurs, the furans and thiophene adopt a mix of parallel and perpendicular π-stacking, according to Figure 3. One such π-stacking occurs between the O21 and the O21^ii^ rings with a distance of 3.674 (3) Å between the planes. Other interactions involve the oxygen or sulfur acting as electron donors while the heterocycles act as electron acceptors. For example, O11î^ interacts with O11—C11—C12—C13—C14, S1 with S1^i^—C16^i^—C17^i^—C18^i^—C19^i^, S2 with S2^ii^—C26^ii^—C27^ii^—C28^ii^—C29^ii^ and O26^ii^ with O26—C211—C212—C213—C214. The centre-to-centre distances for these interactions are 3.517 (3), 3.659 (3), 3.680 (3) and 3.541 (3) Å, respectively.

### Experimental

In 25 ml of anhydrous toluene was added 2-furaldehyde to which was subsequently added DABCO, TiCl_4_ in toluene at 0 °C and then diethyl 2,5-diaminothiophene-3,4-dicarboxylate. The mixture was then refluxed for two hours after which the solvent was removed. Purification by flash chromatography yielded the title product as a red solid. Single crystals of (I) were obtained by slow evaporation of a acetone.

### Refinement

H atoms were placed in calculated positions (C—H = 0.94–0.97 Å) and included in the refinement in the riding-model approximation, with *U*ĩso~(H) = 1.2 *U*~eq~(C).

### Crystal data

**Table:**

C_20_H_18_N_2_O_6_S	*F*_000_ = 1728
*M**_r_* = 414.42	*D*_x_ = 1.425 Mg m^−^^3^
Orthorhombic, *P**c**a*2_1_	Cu *K*α radiation λ = 1.54178 Å
Hall symbol: P 2c -2ac	Cell parameters from 26576 reflections
*a* = 8.2540 (3) Å	θ = 3.8–71.8º
*b* = 10.1578 (3) Å	µ = 1.85 mm^−^^1^
*c* = 46.087 (2) Å	*T* = 220 (2) K
*V* = 3864.0 (2) Å^3^	Block, red
*Z* = 8	0.28 × 0.23 × 0.14 mm

### Data collection

**Table:**

Bruker SMART 2000 diffractometer	7392 independent reflections
Radiation source: X-ray Sealed Tube	7164 reflections with *I* > 2σ(*I*)
Monochromator: graphite	*R*_int_ = 0.030
Detector resolution: 5.5 pixels mm^-1^	θ_max_ = 71.9º
*T* = 220(2) K	θ_min_ = 1.9º
ω scans	*h* = −9→8
Absorption correction: multi-scan(SADABS; Sheldrick, 1996)	*k* = −12→12
*T*_min_ = 0.640, *T*_max_ = 0.781	*l* = −56→55
45562 measured reflections	

### Refinement

**Table:**

Refinement on *F*^2^	Hydrogen site location: inferred from neighbouring sites
Least-squares matrix: full	H-atom parameters constrained
*R*[*F*^2^ > 2σ(*F*^2^)] = 0.032	*w* = 1/[σ^2^(*F*_o_^2^) + (0.0652*P*)^2^ + 0.3926*P*] where *P* = (*F*_o_^2^ + 2*F*_c_^2^)/3
*wR*(*F*^2^) = 0.087	(Δ/σ)_max_ = 0.001
*S* = 1.03	Δρ_max_ = 0.25 e Å^−^^3^
7392 reflections	Δρ_min_ = −0.21 e Å^−^^3^
527 parameters	Extinction correction: none
1 restraint	Absolute structure: Flack (1983), 3551 Friedel pairs
Primary atom site location: structure-invariant direct methods	Flack parameter: 0.021 (10)
Secondary atom site location: difference Fourier map	

### Special details

**Table:**

Experimental. X-ray crystallographic data for I were collected from a single-crystal sample, which was mounted on a loop fiber. Data were collected using a Bruker Platform diffractometer, equipped with a Bruker *SMART* 2 K Charged-Coupled Device (CCD) Area Detector using the program *SMART* and normal focus sealed tube source graphite monochromated Cu—Kα radiation. The crystal-to-detector distance was 4.908 cm, and the data collection was carried out in 512 *x* 512 pixel mode, utilizing 4 *x* 4 pixel binning. The initial unit-cell parameters were determined by a least-squares fit of the angular setting of strong reflections, collected by a 9.0 degree scan in 30 frames over four different parts of the reciprocal space (120 frames total). One complete sphere of data was collected, to better than 0.8Å resolution. Upon completion of the data collection, the first 101 frames were recollected in order to improve the decay correction analysis.
Geometry. All e.s.d.'s (except the e.s.d. in the dihedral angle between two l.s. planes) are estimated using the full covariance matrix. The cell e.s.d.'s are taken into account individually in the estimation of e.s.d.'s in distances, angles and torsion angles; correlations between e.s.d.'s in cell parameters are only used when they are defined by crystal symmetry. An approximate (isotropic) treatment of cell e.s.d.'s is used for estimating e.s.d.'s involving l.s. planes.
Refinement. Refinement of *F*^2^ against ALL reflections. The weighted *R*-factor *wR* and goodness of fit *S* are based on *F*^2^, conventional *R*-factors *R* are based on *F*, with *F* set to zero for negative *F*^2^. The threshold expression of *F*^2^ > σ(*F*^2^) is used only for calculating *R*-factors(gt) *etc*. and is not relevant to the choice of reflections for refinement. *R*-factors based on *F*^2^ are statistically about twice as large as those based on *F*, and *R*- factors based on ALL data will be even larger.

### Fractional atomic coordinates and isotropic or equivalent isotropic displacement parameters (Å^2^)

**Table:**

	*x*	*y*	*z*	*U*_iso_*/*U*_eq_	
S1	0.88387 (5)	0.03066 (4)	0.280712 (9)	0.02929 (11)	
O11	0.7551 (2)	0.00706 (16)	0.39480 (3)	0.0514 (4)	
O12	0.62152 (17)	0.36759 (14)	0.33348 (3)	0.0384 (3)	
O13	0.42379 (16)	0.28718 (14)	0.30508 (3)	0.0378 (3)	
O14	0.61655 (17)	0.44246 (12)	0.26116 (3)	0.0382 (3)	
O15	0.5786 (2)	0.31388 (17)	0.22228 (3)	0.0510 (4)	
O16	0.99383 (17)	0.13629 (12)	0.16959 (3)	0.0347 (3)	
N11	0.76907 (19)	0.08247 (14)	0.33642 (3)	0.0295 (3)	
N12	0.86068 (18)	0.13026 (14)	0.22505 (3)	0.0271 (3)	
C11	0.7720 (4)	−0.0574 (2)	0.42031 (5)	0.0579 (7)	
H11	0.7177	−0.0343	0.4375	0.069*	
C12	0.8754 (3)	−0.1583 (2)	0.41805 (5)	0.0496 (6)	
H12	0.9055	−0.2177	0.4327	0.060*	
C13	0.9302 (3)	−0.1569 (2)	0.38902 (5)	0.0444 (5)	
H13	1.0052	−0.2151	0.3806	0.053*	
C14	0.8537 (2)	−0.0556 (2)	0.37561 (4)	0.0330 (4)	
C15	0.8600 (2)	−0.01021 (18)	0.34629 (5)	0.0307 (4)	
H15	0.9342	−0.0501	0.3336	0.037*	
C16	0.7747 (2)	0.11598 (16)	0.30753 (4)	0.0255 (3)	
C17	0.6861 (2)	0.21581 (15)	0.29546 (3)	0.0236 (3)	
C18	0.7036 (2)	0.22618 (15)	0.26477 (3)	0.0241 (3)	
C19	0.8078 (2)	0.13381 (15)	0.25344 (3)	0.0252 (3)	
C110	0.9376 (2)	0.02961 (17)	0.21527 (4)	0.0290 (4)	
H110	0.9487	−0.0449	0.2272	0.035*	
C111	1.0068 (2)	0.02703 (17)	0.18691 (4)	0.0280 (4)	
C112	1.0949 (2)	−0.06749 (19)	0.17307 (4)	0.0360 (4)	
H112	1.1213	−0.1514	0.1803	0.043*	
C113	1.1388 (3)	−0.0152 (2)	0.14591 (5)	0.0391 (4)	
H113	1.2003	−0.0572	0.1315	0.047*	
C114	1.0754 (3)	0.1067 (2)	0.14468 (4)	0.0378 (4)	
H114	1.0859	0.1640	0.1288	0.045*	
C115	0.5773 (2)	0.30049 (16)	0.31350 (4)	0.0249 (3)	
C116	0.3026 (3)	0.3701 (2)	0.31926 (6)	0.0494 (6)	
H11A	0.2905	0.4532	0.3087	0.059*	
H11B	0.3363	0.3901	0.3392	0.059*	
C117	0.1467 (3)	0.2974 (2)	0.31946 (6)	0.0492 (5)	
H11C	0.1238	0.2647	0.3001	0.074*	
H11D	0.0604	0.3560	0.3256	0.074*	
H11E	0.1539	0.2239	0.3328	0.074*	
C118	0.6262 (2)	0.32909 (17)	0.24645 (4)	0.0278 (4)	
C119	0.5416 (3)	0.55525 (19)	0.24660 (6)	0.0458 (5)	
H11F	0.4718	0.6025	0.2603	0.055*	
H11G	0.4742	0.5243	0.2305	0.055*	
C120	0.6692 (3)	0.6460 (3)	0.23539 (7)	0.0606 (7)	
H12A	0.7376	0.6745	0.2513	0.091*	
H12B	0.6184	0.7220	0.2265	0.091*	
H12C	0.7346	0.6003	0.2211	0.091*	
S2	1.11876 (5)	0.47091 (4)	0.029580 (9)	0.02897 (11)	
O21	1.2717 (2)	0.38213 (14)	0.13750 (3)	0.0462 (3)	
O22	0.8366 (2)	0.17631 (14)	0.08863 (3)	0.0453 (4)	
O23	0.85661 (17)	0.05444 (12)	0.04810 (3)	0.0319 (3)	
O24	0.66327 (15)	0.21385 (13)	0.00495 (3)	0.0335 (3)	
O25	0.86107 (17)	0.12997 (13)	−0.02296 (3)	0.0367 (3)	
O26	0.9906 (2)	0.49263 (17)	−0.08447 (3)	0.0505 (4)	
N21	1.10411 (18)	0.37203 (15)	0.08486 (3)	0.0288 (3)	
N22	1.00539 (18)	0.41810 (15)	−0.02615 (3)	0.0292 (3)	
C21	1.3475 (3)	0.4236 (3)	0.16214 (5)	0.0555 (6)	
H21	1.3902	0.3669	0.1763	0.067*	
C22	1.3536 (3)	0.5541 (3)	0.16356 (5)	0.0564 (6)	
H22	1.3983	0.6050	0.1786	0.068*	
C23	1.2795 (3)	0.6011 (2)	0.13803 (5)	0.0498 (6)	
H23	1.2654	0.6895	0.1325	0.060*	
C24	1.2323 (3)	0.49250 (19)	0.12296 (4)	0.0343 (4)	
C25	1.1536 (2)	0.48230 (18)	0.09525 (4)	0.0329 (4)	
H25	1.1377	0.5593	0.0843	0.039*	
C26	1.0469 (2)	0.36631 (16)	0.05677 (4)	0.0260 (3)	
C27	0.9456 (2)	0.27116 (15)	0.04542 (4)	0.0233 (3)	
C28	0.92640 (19)	0.28218 (16)	0.01478 (3)	0.0236 (3)	
C29	1.0115 (2)	0.38413 (16)	0.00280 (4)	0.0256 (3)	
C210	1.0948 (2)	0.51116 (18)	−0.03598 (4)	0.0302 (4)	
H210	1.1678	0.5520	−0.0232	0.036*	
C211	1.0894 (2)	0.55595 (19)	−0.06526 (4)	0.0323 (4)	
C212	1.1659 (3)	0.6574 (2)	−0.07884 (4)	0.0424 (5)	
H212	1.2407	0.7160	−0.0704	0.051*	
C213	1.1120 (3)	0.6582 (2)	−0.10780 (5)	0.0482 (6)	
H213	1.1429	0.7172	−0.1225	0.058*	
C214	1.0072 (4)	0.5572 (2)	−0.11012 (5)	0.0576 (7)	
H214	0.9525	0.5343	−0.1273	0.069*	
C215	0.8737 (2)	0.16534 (16)	0.06346 (4)	0.0257 (3)	
C216	0.7854 (2)	−0.05829 (18)	0.06304 (5)	0.0363 (4)	
H21A	0.7214	−0.1101	0.0492	0.044*	
H21B	0.7124	−0.0270	0.0783	0.044*	
C217	0.9139 (3)	−0.1440 (2)	0.07620 (6)	0.0536 (6)	
H21C	0.9851	−0.1763	0.0610	0.080*	
H21D	0.8635	−0.2178	0.0860	0.080*	
H21E	0.9764	−0.0931	0.0901	0.080*	
C218	0.8169 (2)	0.19833 (15)	−0.00340 (3)	0.0249 (3)	
C219	0.5416 (2)	0.1321 (2)	−0.00932 (5)	0.0455 (5)	
H21F	0.5309	0.0478	0.0009	0.055*	
H21G	0.5738	0.1143	−0.0294	0.055*	
C220	0.3842 (2)	0.2042 (2)	−0.00878 (6)	0.0467 (6)	
H22A	0.3669	0.2411	0.0104	0.070*	
H22B	0.2968	0.1438	−0.0133	0.070*	
H22C	0.3865	0.2745	−0.0230	0.070*	

### Atomic displacement parameters (Å^2^)

**Table:**

	*U*^11^	*U*^22^	*U*^33^	*U*^12^	*U*^13^	*U*^23^
S1	0.0359 (2)	0.0292 (2)	0.0227 (2)	0.00906 (15)	0.00331 (16)	0.00053 (17)
O11	0.0749 (11)	0.0499 (8)	0.0293 (8)	0.0218 (8)	0.0123 (7)	0.0054 (7)
O12	0.0412 (8)	0.0407 (7)	0.0332 (7)	0.0033 (6)	−0.0031 (5)	−0.0142 (6)
O13	0.0291 (6)	0.0407 (7)	0.0436 (7)	0.0044 (5)	0.0006 (6)	−0.0180 (6)
O14	0.0474 (8)	0.0238 (6)	0.0435 (8)	0.0055 (5)	−0.0125 (6)	−0.0008 (6)
O15	0.0705 (10)	0.0548 (9)	0.0277 (7)	0.0281 (8)	−0.0133 (7)	−0.0079 (6)
O16	0.0452 (8)	0.0314 (6)	0.0277 (7)	0.0046 (5)	0.0076 (5)	0.0025 (5)
N11	0.0363 (8)	0.0294 (7)	0.0229 (7)	0.0022 (6)	0.0018 (6)	0.0018 (6)
N12	0.0308 (7)	0.0287 (7)	0.0218 (7)	0.0014 (5)	0.0027 (5)	−0.0020 (6)
C11	0.0882 (19)	0.0577 (13)	0.0278 (11)	0.0163 (13)	0.0123 (11)	0.0064 (10)
C12	0.0674 (16)	0.0512 (13)	0.0302 (11)	0.0067 (10)	−0.0023 (9)	0.0112 (10)
C13	0.0561 (13)	0.0453 (11)	0.0317 (10)	0.0118 (10)	−0.0023 (9)	0.0055 (9)
C14	0.0394 (10)	0.0345 (9)	0.0251 (9)	0.0046 (7)	0.0006 (7)	−0.0008 (8)
C15	0.0359 (10)	0.0308 (9)	0.0255 (11)	0.0034 (7)	0.0014 (7)	−0.0008 (7)
C16	0.0283 (8)	0.0256 (7)	0.0228 (7)	−0.0006 (6)	0.0029 (6)	−0.0028 (6)
C17	0.0267 (8)	0.0214 (7)	0.0228 (7)	−0.0031 (6)	0.0009 (6)	−0.0014 (6)
C18	0.0274 (8)	0.0228 (7)	0.0222 (7)	−0.0016 (6)	0.0013 (6)	−0.0013 (6)
C19	0.0305 (9)	0.0229 (7)	0.0222 (8)	−0.0004 (6)	0.0009 (6)	0.0010 (6)
C110	0.0358 (10)	0.0293 (9)	0.0221 (8)	0.0045 (7)	0.0013 (7)	0.0002 (6)
C111	0.0325 (9)	0.0284 (9)	0.0230 (8)	0.0036 (7)	0.0001 (6)	−0.0006 (7)
C112	0.0427 (10)	0.0368 (9)	0.0285 (9)	0.0107 (8)	0.0003 (7)	−0.0026 (8)
C113	0.0453 (11)	0.0483 (11)	0.0237 (10)	0.0086 (9)	0.0058 (7)	−0.0044 (8)
C114	0.0488 (11)	0.0420 (10)	0.0225 (8)	0.0008 (9)	0.0058 (7)	0.0031 (7)
C115	0.0322 (9)	0.0214 (7)	0.0212 (8)	−0.0020 (6)	0.0034 (7)	0.0004 (6)
C116	0.0347 (12)	0.0512 (12)	0.0623 (14)	0.0084 (9)	0.0072 (9)	−0.0253 (11)
C117	0.0364 (11)	0.0559 (13)	0.0555 (14)	0.0069 (9)	0.0085 (9)	−0.0017 (11)
C118	0.0263 (9)	0.0296 (8)	0.0275 (9)	0.0041 (7)	0.0036 (6)	−0.0011 (7)
C119	0.0457 (12)	0.0297 (9)	0.0621 (14)	0.0098 (8)	−0.0105 (10)	0.0054 (9)
C120	0.0605 (15)	0.0490 (13)	0.0724 (17)	0.0167 (11)	0.0151 (13)	0.0281 (12)
S2	0.0361 (2)	0.0274 (2)	0.0234 (2)	−0.00820 (15)	−0.00294 (16)	0.00184 (16)
O21	0.0663 (10)	0.0405 (7)	0.0319 (7)	0.0001 (7)	−0.0103 (7)	−0.0015 (6)
O22	0.0677 (10)	0.0425 (8)	0.0257 (7)	−0.0186 (7)	0.0124 (6)	−0.0059 (6)
O23	0.0440 (7)	0.0229 (5)	0.0289 (6)	−0.0054 (5)	0.0046 (5)	−0.0007 (5)
O24	0.0275 (6)	0.0335 (6)	0.0397 (7)	−0.0028 (5)	−0.0026 (5)	−0.0114 (5)
O25	0.0410 (8)	0.0378 (7)	0.0312 (7)	−0.0018 (6)	0.0029 (5)	−0.0119 (6)
O26	0.0727 (11)	0.0496 (8)	0.0292 (8)	−0.0227 (8)	−0.0116 (7)	0.0065 (6)
N21	0.0352 (8)	0.0284 (7)	0.0226 (7)	−0.0022 (6)	−0.0017 (5)	−0.0007 (6)
N22	0.0351 (8)	0.0300 (7)	0.0227 (7)	−0.0028 (6)	−0.0010 (6)	0.0026 (6)
C21	0.0706 (16)	0.0680 (15)	0.0279 (11)	0.0011 (12)	−0.0118 (9)	0.0040 (10)
C22	0.0795 (17)	0.0611 (14)	0.0287 (11)	−0.0281 (13)	−0.0110 (10)	−0.0050 (10)
C23	0.0813 (17)	0.0374 (10)	0.0307 (10)	−0.0234 (11)	−0.0055 (10)	−0.0010 (8)
C24	0.0452 (11)	0.0330 (9)	0.0246 (9)	−0.0095 (8)	0.0010 (7)	−0.0002 (7)
C25	0.0433 (10)	0.0300 (9)	0.0253 (9)	−0.0031 (7)	−0.0013 (8)	−0.0007 (7)
C26	0.0297 (9)	0.0240 (7)	0.0243 (8)	0.0018 (6)	−0.0007 (6)	0.0007 (6)
C27	0.0262 (8)	0.0206 (7)	0.0230 (8)	0.0027 (6)	−0.0004 (6)	−0.0013 (6)
C28	0.0256 (8)	0.0222 (7)	0.0229 (8)	0.0028 (6)	0.0005 (6)	−0.0012 (6)
C29	0.0292 (8)	0.0241 (8)	0.0236 (8)	0.0007 (6)	−0.0017 (6)	0.0001 (6)
C210	0.0370 (10)	0.0302 (8)	0.0233 (10)	−0.0031 (7)	−0.0005 (8)	−0.0008 (7)
C211	0.0396 (10)	0.0320 (9)	0.0252 (9)	−0.0047 (7)	0.0008 (7)	−0.0002 (8)
C212	0.0553 (12)	0.0416 (11)	0.0303 (10)	−0.0136 (9)	0.0025 (9)	0.0051 (8)
C213	0.0665 (15)	0.0490 (13)	0.0292 (10)	−0.0076 (10)	0.0045 (9)	0.0123 (9)
C214	0.0891 (18)	0.0600 (14)	0.0237 (10)	−0.0137 (13)	−0.0135 (10)	0.0078 (10)
C215	0.0282 (9)	0.0270 (8)	0.0219 (8)	−0.0007 (6)	0.0000 (6)	−0.0005 (6)
C216	0.0374 (10)	0.0292 (9)	0.0423 (10)	−0.0100 (7)	0.0035 (8)	0.0040 (8)
C217	0.0505 (13)	0.0393 (12)	0.0711 (17)	−0.0130 (10)	−0.0103 (12)	0.0223 (11)
C218	0.0308 (9)	0.0211 (7)	0.0227 (8)	0.0003 (6)	−0.0029 (7)	0.0038 (6)
C219	0.0321 (10)	0.0445 (11)	0.0597 (14)	−0.0065 (8)	−0.0065 (9)	−0.0176 (10)
C220	0.0333 (11)	0.0522 (13)	0.0547 (14)	−0.0043 (8)	−0.0070 (9)	0.0035 (11)

### Geometric parameters (Å, °)

**Table:**

S1—C19	1.7528 (16)	S2—C26	1.7468 (17)
S1—C16	1.7579 (16)	S2—C29	1.7563 (17)
O11—C11	1.353 (3)	O21—C24	1.346 (2)
O11—C14	1.360 (2)	O21—C21	1.363 (3)
O12—C115	1.202 (2)	O22—C215	1.205 (2)
O13—C115	1.332 (2)	O23—C215	1.338 (2)
O13—C116	1.462 (2)	O23—C216	1.460 (2)
O14—C118	1.339 (2)	O24—C218	1.334 (2)
O14—C119	1.465 (2)	O24—C219	1.460 (2)
O15—C118	1.191 (2)	O25—C218	1.195 (2)
O16—C114	1.364 (2)	O26—C214	1.359 (3)
O16—C111	1.371 (2)	O26—C211	1.365 (2)
N11—C15	1.287 (2)	N21—C25	1.285 (2)
N11—C16	1.375 (2)	N21—C26	1.379 (2)
N12—C110	1.285 (2)	N22—C210	1.282 (2)
N12—C19	1.380 (2)	N22—C29	1.379 (2)
C11—C12	1.338 (3)	C21—C22	1.329 (4)
C11—H11	0.94	C21—H21	0.94
C12—C13	1.412 (3)	C22—C23	1.410 (3)
C12—H12	0.94	C22—H22	0.94
C13—C14	1.356 (3)	C23—C24	1.361 (3)
C13—H13	0.94	C23—H23	0.94
C14—C15	1.429 (3)	C24—C25	1.437 (3)
C15—H15	0.94	C25—H25	0.94
C16—C17	1.368 (2)	C26—C27	1.380 (2)
C17—C18	1.426 (2)	C27—C28	1.425 (2)
C17—C115	1.496 (2)	C27—C215	1.483 (2)
C18—C19	1.376 (2)	C28—C29	1.368 (2)
C18—C118	1.488 (2)	C28—C218	1.498 (2)
C110—C111	1.426 (2)	C210—C211	1.425 (3)
C110—H110	0.94	C210—H210	0.94
C111—C112	1.363 (2)	C211—C212	1.361 (3)
C112—C113	1.407 (3)	C212—C213	1.407 (3)
C112—H112	0.94	C212—H212	0.94
C113—C114	1.346 (3)	C213—C214	1.347 (3)
C113—H113	0.94	C213—H213	0.94
C114—H114	0.94	C214—H214	0.94
C116—C117	1.484 (3)	C216—C217	1.500 (3)
C116—H11a	0.98	C216—H21a	0.98
C116—H11b	0.98	C216—H21b	0.98
C117—H11c	0.97	C217—H21c	0.97
C117—H11d	0.97	C217—H21d	0.97
C117—H11e	0.97	C217—H21e	0.97
C119—C120	1.492 (3)	C219—C220	1.491 (3)
C119—H11f	0.98	C219—H21f	0.98
C119—H11g	0.98	C219—H21g	0.98
C120—H12a	0.97	C220—H22a	0.97
C120—H12b	0.97	C220—H22b	0.97
C120—H12c	0.97	C220—H22c	0.97
			
C19—S1—C16	91.48 (8)	C26—S2—C29	91.57 (8)
C11—O11—C14	106.08 (17)	C24—O21—C21	105.59 (17)
C115—O13—C116	117.53 (15)	C215—O23—C216	116.98 (14)
C118—O14—C119	117.79 (16)	C218—O24—C219	117.17 (14)
C114—O16—C111	105.86 (14)	C214—O26—C211	106.05 (17)
C15—N11—C16	120.24 (16)	C25—N21—C26	119.69 (16)
C110—N12—C19	120.65 (15)	C210—N22—C29	120.37 (16)
C12—C11—O11	111.6 (2)	C22—C21—O21	111.5 (2)
C12—C11—H11	124.2	C22—C21—H21	124.3
O11—C11—H11	124.2	O21—C21—H21	124.3
C11—C12—C13	105.7 (2)	C21—C22—C23	106.3 (2)
C11—C12—H12	127.2	C21—C22—H22	126.8
C13—C12—H12	127.2	C23—C22—H22	126.8
C14—C13—C12	106.9 (2)	C24—C23—C22	106.0 (2)
C14—C13—H13	126.6	C24—C23—H23	127
C12—C13—H13	126.6	C22—C23—H23	127
C13—C14—O11	109.71 (18)	O21—C24—C23	110.61 (17)
C13—C14—C15	131.22 (19)	O21—C24—C25	119.47 (17)
O11—C14—C15	119.06 (17)	C23—C24—C25	129.91 (19)
N11—C15—C14	123.32 (18)	N21—C25—C24	122.54 (17)
N11—C15—H15	118.3	N21—C25—H25	118.7
C14—C15—H15	118.3	C24—C25—H25	118.7
C17—C16—N11	123.99 (15)	N21—C26—C27	126.33 (15)
C17—C16—S1	110.70 (13)	N21—C26—S2	122.09 (13)
N11—C16—S1	125.17 (13)	C27—C26—S2	111.08 (12)
C16—C17—C18	113.83 (15)	C26—C27—C28	112.81 (15)
C16—C17—C115	121.40 (15)	C26—C27—C215	122.50 (15)
C18—C17—C115	124.73 (15)	C28—C27—C215	124.60 (15)
C19—C18—C17	112.93 (15)	C29—C28—C27	113.72 (15)
C19—C18—C118	122.16 (15)	C29—C28—C218	120.97 (15)
C17—C18—C118	124.84 (15)	C27—C28—C218	125.21 (15)
C18—C19—N12	125.14 (15)	C28—C29—N22	124.16 (15)
C18—C19—S1	111.07 (12)	C28—C29—S2	110.81 (13)
N12—C19—S1	123.44 (13)	N22—C29—S2	124.93 (13)
N12—C110—C111	122.27 (16)	N22—C210—C211	123.53 (18)
N12—C110—H110	118.9	N22—C210—H210	118.2
C111—C110—H110	118.9	C211—C210—H210	118.2
C112—C111—O16	109.84 (16)	C212—C211—O26	109.61 (17)
C112—C111—C110	130.94 (17)	C212—C211—C210	131.53 (19)
O16—C111—C110	119.16 (15)	O26—C211—C210	118.85 (17)
C111—C112—C113	106.72 (17)	C211—C212—C213	107.10 (19)
C111—C112—H112	126.6	C211—C212—H212	126.5
C113—C112—H112	126.6	C213—C212—H212	126.5
C114—C113—C112	106.54 (17)	C214—C213—C212	105.89 (19)
C114—C113—H113	126.7	C214—C213—H213	127.1
C112—C113—H113	126.7	C212—C213—H213	127.1
C113—C114—O16	111.04 (17)	C213—C214—O26	111.34 (19)
C113—C114—H114	124.5	C213—C214—H214	124.3
O16—C114—H114	124.5	O26—C214—H214	124.3
O12—C115—O13	124.72 (16)	O22—C215—O23	124.07 (16)
O12—C115—C17	124.69 (17)	O22—C215—C27	125.07 (16)
O13—C115—C17	110.55 (14)	O23—C215—C27	110.86 (14)
O13—C116—C117	108.03 (17)	O23—C216—C217	111.14 (16)
O13—C116—H11A	110.1	O23—C216—H21A	109.4
C117—C116—H11A	110.1	C217—C216—H21A	109.4
O13—C116—H11B	110.1	O23—C216—H21B	109.4
C117—C116—H11B	110.1	C217—C216—H21B	109.4
H11A—C116—H11B	108.4	H21A—C216—H21B	108
C116—C117—H11C	109.5	C216—C217—H21C	109.5
C116—C117—H11D	109.5	C216—C217—H21D	109.5
H11C—C117—H11D	109.5	H21C—C217—H21D	109.5
C116—C117—H11E	109.5	C216—C217—H21E	109.5
H11C—C117—H11E	109.5	H21C—C217—H21E	109.5
H11D—C117—H11E	109.5	H21D—C217—H21E	109.5
O15—C118—O14	124.43 (17)	O25—C218—O24	125.20 (16)
O15—C118—C18	125.53 (16)	O25—C218—C28	124.60 (16)
O14—C118—C18	110.04 (14)	O24—C218—C28	110.19 (14)
O14—C119—C120	110.11 (18)	O24—C219—C220	108.20 (17)
O14—C119—H11F	109.6	O24—C219—H21F	110.1
C120—C119—H11F	109.6	C220—C219—H21F	110.1
O14—C119—H11G	109.6	O24—C219—H21G	110.1
C120—C119—H11G	109.6	C220—C219—H21G	110.1
H11F—C119—H11G	108.2	H21F—C219—H21G	108.4
C119—C120—H12A	109.5	C219—C220—H22A	109.5
C119—C120—H12B	109.5	C219—C220—H22B	109.5
H12A—C120—H12B	109.5	H22A—C220—H22B	109.5
C119—C120—H12C	109.5	C219—C220—H22C	109.5
H12A—C120—H12C	109.5	H22A—C220—H22C	109.5
H12B—C120—H12C	109.5	H22B—C220—H22C	109.5
			
C14—O11—C11—C12	−0.5 (3)	C24—O21—C21—C22	1.1 (3)
O11—C11—C12—C13	0.8 (3)	O21—C21—C22—C23	−1.0 (3)
C11—C12—C13—C14	−0.8 (3)	C21—C22—C23—C24	0.5 (3)
C12—C13—C14—O11	0.5 (3)	C21—O21—C24—C23	−0.8 (3)
C12—C13—C14—C15	−178.5 (2)	C21—O21—C24—C25	178.3 (2)
C11—O11—C14—C13	0.0 (3)	C22—C23—C24—O21	0.2 (3)
C11—O11—C14—C15	179.1 (2)	C22—C23—C24—C25	−178.8 (2)
C16—N11—C15—C14	−176.15 (18)	C26—N21—C25—C24	−172.38 (17)
C13—C14—C15—N11	174.4 (2)	O21—C24—C25—N21	7.1 (3)
O11—C14—C15—N11	−4.5 (3)	C23—C24—C25—N21	−173.9 (2)
C15—N11—C16—C17	−177.87 (18)	C25—N21—C26—C27	−158.36 (18)
C15—N11—C16—S1	6.9 (2)	C25—N21—C26—S2	30.5 (2)
C19—S1—C16—C17	0.44 (13)	C29—S2—C26—N21	172.08 (15)
C19—S1—C16—N11	176.23 (15)	C29—S2—C26—C27	−0.28 (13)
N11—C16—C17—C18	−175.90 (15)	N21—C26—C27—C28	−171.76 (16)
S1—C16—C17—C18	−0.05 (19)	S2—C26—C27—C28	0.21 (18)
N11—C16—C17—C115	1.8 (3)	N21—C26—C27—C215	5.0 (3)
S1—C16—C17—C115	177.66 (12)	S2—C26—C27—C215	176.96 (12)
C16—C17—C18—C19	−0.5 (2)	C26—C27—C28—C29	0.0 (2)
C115—C17—C18—C19	−178.13 (15)	C215—C27—C28—C29	−176.66 (15)
C16—C17—C18—C118	−177.43 (15)	C26—C27—C28—C218	−176.23 (15)
C115—C17—C18—C118	4.9 (3)	C215—C27—C28—C218	7.1 (3)
C17—C18—C19—N12	−172.48 (16)	C27—C28—C29—N22	−176.66 (15)
C118—C18—C19—N12	4.5 (3)	C218—C28—C29—N22	−0.3 (2)
C17—C18—C19—S1	0.83 (18)	C27—C28—C29—S2	−0.21 (18)
C118—C18—C19—S1	177.85 (13)	C218—C28—C29—S2	176.19 (12)
C110—N12—C19—C18	−169.26 (17)	C210—N22—C29—C28	−176.66 (17)
C110—N12—C19—S1	18.2 (2)	C210—N22—C29—S2	7.4 (2)
C16—S1—C19—C18	−0.72 (13)	C26—S2—C29—C28	0.28 (13)
C16—S1—C19—N12	172.72 (15)	C26—S2—C29—N22	176.70 (15)
C19—N12—C110—C111	−174.25 (16)	C29—N22—C210—C211	−176.86 (17)
C114—O16—C111—C112	0.3 (2)	C214—O26—C211—C212	−0.2 (3)
C114—O16—C111—C110	177.77 (17)	C214—O26—C211—C210	178.7 (2)
N12—C110—C111—C112	178.2 (2)	N22—C210—C211—C212	175.0 (2)
N12—C110—C111—O16	1.3 (3)	N22—C210—C211—O26	−3.6 (3)
O16—C111—C112—C113	−0.1 (2)	O26—C211—C212—C213	0.3 (3)
C110—C111—C112—C113	−177.2 (2)	C210—C211—C212—C213	−178.4 (2)
C111—C112—C113—C114	−0.1 (2)	C211—C212—C213—C214	−0.3 (3)
C112—C113—C114—O16	0.3 (3)	C212—C213—C214—O26	0.2 (3)
C111—O16—C114—C113	−0.4 (2)	C211—O26—C214—C213	0.0 (3)
C116—O13—C115—O12	6.4 (3)	C216—O23—C215—O22	1.2 (3)
C116—O13—C115—C17	−176.02 (17)	C216—O23—C215—C27	−179.36 (15)
C16—C17—C115—O12	60.1 (2)	C26—C27—C215—O22	32.2 (3)
C18—C17—C115—O12	−122.5 (2)	C28—C27—C215—O22	−151.47 (19)
C16—C17—C115—O13	−117.47 (18)	C26—C27—C215—O23	−147.22 (15)
C18—C17—C115—O13	60.0 (2)	C28—C27—C215—O23	29.1 (2)
C115—O13—C116—C117	−150.0 (2)	C215—O23—C216—C217	−92.5 (2)
C119—O14—C118—O15	0.3 (3)	C219—O24—C218—O25	5.6 (3)
C119—O14—C118—C18	−179.32 (16)	C219—O24—C218—C28	−175.98 (16)
C19—C18—C118—O15	37.5 (3)	C29—C28—C218—O25	63.6 (2)
C17—C18—C118—O15	−145.8 (2)	C27—C28—C218—O25	−120.41 (19)
C19—C18—C118—O14	−142.83 (16)	C29—C28—C218—O24	−114.78 (17)
C17—C18—C118—O14	33.8 (2)	C27—C28—C218—O24	61.2 (2)
C118—O14—C119—C120	−100.7 (2)	C218—O24—C219—C220	−152.24 (18)
